# Leber’s hereditary optic neuropathy – current status of idebenone and gene replacement therapies

**DOI:** 10.1515/medgen-2024-2066

**Published:** 2025-02-12

**Authors:** Thomas Klopstock, Leopold H. Zeng, Claudia Priglinger

**Affiliations:** LMU Munich Friedrich Baur Institute at the Department of Neurology, LMU University Hospital Ziemssenstr. 1 80336 Munich Germany; LMU Munich Friedrich Baur Institute at the Department of Neurology, LMU University Hospital Ziemssenstr. 1 80336 Munich Germany; LMU Munich Department of Ophthalmology, LMU University Hospital Mathildenstr. 8 80336 Munich Germany

**Keywords:** LHON, mtDNA, Complex I, idebenone, gene therapy

## Abstract

Leber’s hereditary optic neuropathy (LHON) is the most common mitochondrial disease, and was the first to be linked to mitochondrial DNA (mtDNA) variations. Recently, autosomal recessive forms of LHON were described in addition to the classical mtDNA-associated forms. Clinically, LHON manifests with subacute and painless loss of central visual acuity, in most cases starting unilaterally, and involving the second eye a few weeks later. Almost all LHON cases are caused by pathogenic variants in genes that code for proteins relevant for function of Complex I of the respiratory chain. The Complex I dysfunction in LHON leads to decreased ATP synthesis and to increased production of reactive oxygen species which ultimately initiates dysfunction and apoptosis of retinal ganglion cells and their axons, the optic nerve. Idebenone, a synthetic CoQ derivative, is a potent intramitochondrial antioxidant and can shuttle electrons directly to complex III of the respiratory chain, thereby bypassing complex I deficiency. On the basis of several clinical trials, it has been approved as a treatment for LHON in 2015 (in the EU). In addition, direct intravitreal gene replacement therapy is being investigated, with several late-stage clinical trials already completed. In the future, gene editing of mtDNA variants may also become a therapeutic option.

## Phenotype and genetics

LHON is the most common mitochondrial disease, affecting about 1 in 31 000 [1] to 1 in 50 000 people [2]. The phenotype of the disorder was first described by Albrecht von Graefe in 1858 and then in more detail by Theodor Leber in 1871 [3]. LHON was also the first disorder ever linked to mitochondrial DNA (mtDNA) variations, in 1988 [4]. The disease primarily affects retinal ganglion cells and their axons (i.e the optic nerve), resulting in significant vision loss and consecutive optic nerve atrophy [5, 6]. Environmental factors, in particular smoking, are important risk factors and can trigger the onset of visual deterioration [7]. This painless loss of vision typically starts in one eye and progresses to the other within weeks to months [8], often leading to legal blindness [9]. Depending on age and genotype, there may be some degree of recovery in a subset of patients but the majority experiences a permanent and severe impact on central visual acuity and quality of life [9].

Most cases of LHON (approximately 90 %) are caused by single missense variants in the mtDNA, in particular m.11778G>A in the mt-*ND4* gene, m.3460G>A in the mt-*ND1* gene and m.14484T>C in the mt-*ND6* gene) (Table 1). Most patients are homoplasmic, i. e. they carry 100 % of the variant in each cell of the body [10]. The mtDNA and accordingly mtDNA-associated LHON is exclusively transmitted through the maternal lineage. Of note, the penetrance of these mtDNA variants is rather low, i.e only a certain proportion of mutation carriers manifests the disease. For the three classic variants combined, the penetrance has recently been calculated to be 17.5 % in males and 5.4 % in females [11], leading to a male-to-female ratio of around 3:1 in affected patients. The penetrance values per mutation and sex are provided in Table 1.

In addition to this mtDNA-associated form of LHON (mtLHON), nuclear gene defects have recently been identified as causes of an autosomal recessive form of LHON (arLHON) [12]. By far the most frequent of these nuclear gene defects is biallelic variants in *DNAJC30*, a single exon gene on chromosome 7 [13, 14]. Despite the different mode of inheritance, *DNAJC30*-associated LHON is clinically near-indistinguishable from mtLHON, and even recapitulates features such as reduced penetrance and male predominance [13]. Another cause of arLHON, so far described in only one family, is biallelic variants in the *NDUFS2* gene [15]. Of note, all of the above LHON gene defects affect Complex I of the respiratory chain. The mtDNA *ND4*, *ND6* and *ND1* genes as well as the nuclear *NDUFS2* gene code for structural subunits of Complex I while the *DNAJC30* gene codes for a chaperone protein involved in maintenance and repair of Complex I (Table 1) [13, 15].

**Table 1:** Molecular causes of LHON

Molecular cause	Pathomechanism	Relative proportion or number of cases	Penetrance (m/f)
**mtDNA**			
m.11778G>A variant in *ND4* gene	ND4 is a subunit of Complex I	69 % [16]	16.2 %/4.3 % [11]
m.14484T>C variant in *ND6* gene	ND6 is a subunit of Complex I	17 % [16]	18.4 %/3.4 % [11]
m.3460G>A variant in *ND1* gene	ND1 is a subunit of Complex I	13 % [16]	18.0 %/5.7 % [11]
**nuclear**			
*DNAJC30* variants	DNAJC30 is involved in maintenance and repair of Complex I [13]	> 90 patients described so far [13, 14, 17, 18]	96.8 %/42.9 % [13]
*NDUFS2* variants [15]	NDUFS2 is a subunit of C1	Only one family described so far	n/a

*Abbreviations: m, male; f, female; mtDNA, mitochondrial DNA; ND, NADH dehydrogenase subunit*

## Pathomechanisms

Complex I is the entry point of electron flow through the mitochondrial respiratory chain and interacts with coenzyme Q to facilitate proton translocation across the inner mitochondrial membrane. Both electron flow and build-up of an electrochemical gradient are ultimately needed to drive mitochondrial energy production in the form of ATP [19].

Complex I deficiency, as caused by the gene defects described above, lead to decreased ATP synthesis and, even more importantly, to increased production of reactive oxygen species (ROS) [5]. Subsequently, ROS and complex I deficiency lead to opening of the mitochondrial permeability transition pore (MPTP) initiating dysfunction and ultimately apoptosis of cells [20, 21].

Why is LHON so tissue-specific and affects (in most cases) only the retinal ganglion cells (RGCs) and their axons, the optic nerve? The photoreceptors convert incoming visual signals into a receptor potential, which is transmitted via the bipolar cells and then processed in the RGCs. The axons of the RGCs (optic nerve) transmit the signal to the brain as action potentials [6]. These axons are partially unmyelinated (until they enter the optic nerve) to maintain the nerve fiber layer’s permeability to light which requires energy-intense continuous instead of energy-saving saltatory conduction, rendering them particularly vulnerable to compromised mitochondrial ATP supply [5, 6]. It is unclear, however, why the RGCs and the optic nerve can remain unaffected in other mitochondrial disorders and even in other forms of Complex I defect.

## Idebenone therapy

Idebenone is a synthetic CoQ derivative with a shorter side chain. It can act as an electron carrier in the respiratory chain to contribute to ATP production and also functions as a potent intramitochondrial antioxidant. Of note, it can shuttle electrons directly to complex III of the respiratory chain, thereby bypassing complex I deficiency which renders it a very attractive treatment option for Complex I defect disorders such as LHON [22].

Following positive anecdotal reports, a randomized study with 900 mg/d idebenone vs. placebo over 24 weeks in 85 patients with LHON showed no significance for the primary endpoint, but consistent trends or significances in secondary endpoints and various subgroups favored efficacy [23]. In a retrospective analysis, 45.5 % of 44 treated LHON patients experienced at least partial vision recovery, compared to only 32.2 % of 59 untreated patients [24]. An Expanded Access Program with open-label treatment of 111 LHON patients with 900 mg/d idebenone showed improved chances of stabilization in those patients with well-preserved vision (in 50 % of patients) or clinically relevant recovery in those with significantly reduced vision. The latter was observed in 46.0 % of treated patients (versus 31.1 % in a historical control group), with an average improvement of more than 7 lines on the eye chart [25]. The totality of study results led to the European approval of idebenone in 2015. A post-approval study confirmed the efficacy of idebenone when initiated in the subacute/dynamic phase (up to 1 year after onset) as well as in the chronic phase (1–5 years after onset) but also showed that the treatment effect varies depending on disease phase and causative mtDNA mutation [26]. In addition, patients with *DNAJC30*-associated arLHON benefit markedly from idebenone as well [13, 18]. Since idebenone needs to be reduced intracellularly by an enzyme called NAD(P)H oxidoreductase 1 (NQO1) to exert its beneficial effects [22, 27], response to idebenone therapy is also dependent on NQO1 activity, particularly in patients with the m.3460G>A variant [28]. (Table 2)

**Table 2:** Studies of Idebenone in LHON

Study	No. of patients	Study design and dosage	Inclusion criteria/Study cohort	Results
Klopstock et al., 2011: *RHODOS study* [23]	85	Prospective, randomized, double-blind, placebo-controlled study; 900mg/day	m.11778G>A-, m.3460G>A- or m.14484T>C-variant; Age between 14 and 64; vision loss due to LHON within 5 years before inclusion	No significant difference after 24 weeks, subgroup analysis showed benefit for m.11778G>A and m.3460G>A
Carelli al., 2011 [24]	103	Retrospective study of treated and untreated patients, varying dosages	Follow-up ≥ 5 years; in treated patients start of idebenone within first year after disease onset	Showed benefit for m.11778G>A-patients; time from disease onset to the start of treatment has been shown to be important
Catarino et al., 2020: Expanded Access Program [25]	111	Retrospective study of treated patients, 900mg/day	m.11778G>A-, m.3460G>A- or m.14484T>C-variant; idebenone therapy started within first year after onset	46 % (40/87) of patients showed clinically relevant recovery
van Everdingen et al., 2022 [29]	72	Retrospective study, 900mg/day	Complex I-affecting variants	56 % (40/72) of patients showed clinically relevant recovery
Yu-Wai-Man et al., 2024: *LEROS study* [26]	199 + 372 (natural history cohort)	Open-label, interventional study, 900mg/day	Patients up to 5 years after onset, treatment period of 2 years	More patients had clinically relevant recovery after 12 and 24 months in comparison to natural history cohort: 47.9 % vs. 33.3 % when treatment was started in subacute/dynamic phase; 31.9 % vs. 16.1 % when treatment was started in chronic phase (24 months data)

## Gene replacement therapy

In addition to the established idebenone therapy, novel gene therapy approaches are being explored. These approaches primarily involve the permanent provision of a functional gene copy to compensate for the defective native gene. Currently, three similar gene therapy vectors based on recombinant adeno-associated viruses (rAAV) are undergoing clinical trials, with studies being conducted in the USA, Europe, and China.

Since direct gene transfer to mitochondria is not yet established in human, the AAV vectors are targeted to the nucleus where the wild-type gene is transcribed into mRNA. The mRNA is then translated at cytosolic ribosomes into protein that, guided by a mitochondrial targeting sequence, can enter mitochondria via their physiological protein import machinery [6].

The most advanced gene therapies target patients carrying the m.11778G>A variant of the mt-*ND4* gene. The rAAV2/2-ND4 vector contains [30] the wild-type *ND4* gene, an upstream mitochondrial targeting sequence required for the transfer of the translated protein across the mitochondrial inner membrane [31], and downstream the 3’-untranslated region (3’-UTR) of the nuclear *COX10* gene which turned out to be advantageous for allotopic expression [30].

Lenadogene nolparvovec, a recombinant adeno-associated virus 2 (rAAV2) vector containing a wildtype version of the *ND4* gene (the whole construct being abbreviated as rAAV2/ND4), was the first compound to be investigated in Phase III clinical trials. In two parallel studies, unilateral injection of the gene therapy vector 0–6 months (RESCUE) [32] or 6–12 months (REVERSE) [33] after onset unexpectedly led to bilateral improvement of visual acuity which was beneficial for the patients but foiled the predefined primary endpoint of both studies. A later study (REFLECT) [8] showed additional benefit of bilateral as compared to unilateral injection of rAAV2/2-ND4.

**Table 3:** Gene therapy studies in LHON

Study	Vector	Study type and design	Patients	Outcomes
Feuer et al., 2016 [34]	scAAV2-P1ND4v2	Phase I, open-label	5 patients received unilateral treatment	No serious systemic adverse events
Guy et al., 2017 [35]	scAAV2-P1ND4v2	Phase I, open-label	14 patients received unilateral treatment	Low and medium dosages proved to be safe
Lam et al., 2022 [36]	scAAV2-P1ND4v2	Phase I, open-label	28 patients received unilateral treatment	Favorable safety and tolerability profile
Wan et al., 2016 [37]	rAAV2-ND4	Not applicable	9 patients	Favorable safety profile
Vignal-Clermont et al., 2021: REVEAL study [38]	Lenadogene nolparvovec	Phase I/IIa open-label, 5 yrs follow-up	15 patients received unilateral treatment	Overall well tolerated, most frequent TEAEs were intraocular inflammation and elevation of intraocular pressure, showed improvement in both eyes (LogMAR), with no significant difference after 5 yrs
Yu-Wai-Man et al., 2020: REVERSE study [33]	Lenadogene nolparvovec	Phase III, randomized, double-blind	37 patients with vision loss for 6–12 months had unilateral vector and contralateral sham injection	Showed CRR in vector-treated and sham-treated eyes (LogMAR) after 48 and 96 weeks, but no significant difference between both eyes
Newman et al., 2021: RESCUE study [32]	Lenadogene nolparvovec	Phase III, randomized, double-blind	39 patients with vision loss < 6 months had unilateral vector and contralateral sham injection	Showed improvement in vector-treated and sham-treated eyes (LogMAR) after 96 weeks, but no significant difference between both eyes
Biousse et al., 2021: RESTORE study [39]	Lenadogene nolparvovec	3–5 yrs follow-up after RESCUE and REVERSE studies	61 patients	Progressive and sustained improvement in 3–5 yrs follow-up
Newman et al., 2023: REFLECT study [8]	Lenadogene nolparvovec	Phase III, randomized, double-blind	48 patients had bilateral vector injection; 50 patients had unilateral vector and contralateral sham injection	Better treatment effect in bilateral than in unilateral vector injection after 1.5 yrs

*Abbreviations: yrs, years; TEAEs, treatment-emergent adverse events; CRR, clinically relevant response*

All gene therapy studies in LHON so far are summarized in Table 3. However, as of July 2024, no gene therapy approach has been approved for LHON by regulatory authorities in the USA or the EU.

## Further treatment approaches in development

Gene replacement therapies targeting the m.3460G>A variant have also demonstrated promising results in mouse experiments [40]. Currently, a gene therapy vector targeting the m.3460G>A variant is investigated in a Phase I/II clinical trial (ClinicalTrials.gov ID: NCT05820152).

In the future, gene editing of mtDNA variants may become a viable therapeutic option. While the application of the CRISPR/Cas9 system is limited due to the difficulty of importing guide RNA into mitochondria [41], CRISPR-free mitochondrial base editing is currently under preclinical investigation [42–44], and may have broad implications for the future treatment of mitochondrial disorders.

## References

[1] Yu-Wai-Man P, Griffiths PG, Brown DT, Howell N, Turnbull DM, Chinnery PF (2003). The epidemiology of Leber hereditary optic neuropathy in the North East of England. Am J Hum Genet;72(2).

[2] Puomila A, Hämäläinen P, Kivioja S, Savontaus M-L, Koivumäki S, Huoponen K, Nikoskelainen E (2007). Epidemiology and penetrance of Leber hereditary optic neuropathy in Finland. European Journal of Human Genetics;15(10).

[3] Leber T (1871). Ueber hereditäre und congenital-angelegte Sehnervenleiden. Albrecht von Graefes Archiv für Ophthalmologie;17(2).

[4] Wallace DC, Singh G, Lott MT, Hodge JA, Schurr TG, Lezza AMS, Elsas LJ, Nikoskelainen EK (1988). Mitochondrial DNA Mutation Associated with Leber’s Hereditary Optic Neuropathy. Science;242(4884).

[5] Amore G, Romagnoli M, Carbonelli M, Barboni P, Carelli V, La Morgia C (2021). Therapeutic Options in Hereditary Optic Neuropathies. Drugs;81(1).

[6] Shamsnajafabadi H, MacLaren RE, Cehajic-Kapetanovic J (2023). Current and Future Landscape in Genetic Therapies for Leber Hereditary Optic Neuropathy. Cells;12(15).

[7] Kirkman MA, Yu-Wai-Man P, Korsten A, Leonhardt M, Dimitriadis K, De Coo IF, Klopstock T, Chinnery PF (2009). Gene-environment interactions in Leber hereditary optic neuropathy. Brain;132(Pt 9).

[8] Newman NJ, Yu-Wai-Man P, Subramanian PS, Moster ML, Wang AG, Donahue SP, Leroy BP, Carelli V, Biousse V, Vignal-Clermont C, Sergott RC, Sadun AA, Rebolleda Fernández G, Chwalisz BK, Banik R, Bazin F, Roux M, Cox ED, Taiel M, Sahel JA (2023). Randomized trial of bilateral gene therapy injection for m.11778G>A MT-ND4 Leber optic neuropathy. Brain;146(4).

[9] Kirkman MA, Korsten A, Leonhardt M, Dimitriadis K, De Coo IF, Klopstock T, Griffiths PG, Hudson G, Chinnery PF, Yu-Wai-Man P (2009). Quality of Life in Patients with Leber Hereditary Optic Neuropathy. Investigative Ophthalmology & Visual Science;50(7).

[10] Baglivo M, Nasca A, Lamantea E, Vinci S, Spagnolo M, Marchet S, Prokisch H, Catania A, Lamperti C, Ghezzi D (2023). Evaluation of Mitochondrial Dysfunction and Idebenone Responsiveness in Fibroblasts from Leber’s Hereditary Optic Neuropathy (LHON) Subjects. Int J Mol Sci;24(16).

[11] Lopez Sanchez MIG, Kearns LS, Staffieri SE, Clarke L, McGuinness MB, Meteoukki W, Samuel S, Ruddle JB, Chen C, Fraser CL, Harrison J, Hewitt AW, Howell N, Mackey DA (2021). Establishing risk of vision loss in Leber hereditary optic neuropathy. Am J Hum Genet;108(11).

[12] Lenaers G, Beaulieu C, Charif M, Gerber S, Kaplan J, Rozet JM (2023). Autosomal recessive Leber hereditary optic neuropathy, a new neuro-ophthalmo-genetic paradigm. Brain;146(8).

[13] Stenton SL, Sheremet NL, Catarino CB, Andreeva NA, Assouline Z, Barboni P, Barel O, Berutti R, Bychkov I, Caporali L, Capristo M, Carbonelli M, Cascavilla ML, Charbel Issa P, Freisinger P, Gerber S, Ghezzi D, Graf E, Heidler J, Hempel M, Heon E, Itkis YS, Javasky E, Kaplan J, Kopajtich R, Kornblum C, Kovacs-Nagy R, Krylova TD, Kunz WS, La Morgia C, Lamperti C, Ludwig C, Malacarne PF, Maresca A, Mayr JA, Meisterknecht J, Nevinitsyna TA, Palombo F, Pode-Shakked B, Shmelkova MS, Strom TM, Tagliavini F, Tzadok M, van der Ven AT, Vignal-Clermont C, Wagner M, Zakharova EY, Zhorzholadze NV, Rozet JM, Carelli V, Tsygankova PG, Klopstock T, Wittig I, Prokisch H (2021). Impaired complex I repair causes recessive Leber’s hereditary optic neuropathy. J Clin Invest;131(6).

[14] Kieninger S, Xiao T, Weisschuh N, Kohl S, Rüther K, Kroisel PM, Brockmann T, Knappe S, Kellner U, Lagrèze W, Mazzola P, Haack TB, Wissinger B, Tonagel F (2022). DNAJC30 disease-causing gene variants in a large Central European cohort of patients with suspected Leber’s hereditary optic neuropathy and optic atrophy. J Med Genet;59(10).

[15] Gerber S, Ding MG, Gérard X, Zwicker K, Zanlonghi X, Rio M, Serre V, Hanein S, Munnich A, Rotig A, Bianchi L, Amati-Bonneau P, Elpeleg O, Kaplan J, Brandt U, Rozet J-M (2017). Compound heterozygosity for severe and hypomorphic <em>NDUFS2</em> mutations cause non-syndromic LHON-like optic neuropathy. Journal of Medical Genetics;54(5).

[16] Poincenot L, Pearson AL, Karanjia R (2020). Demographics of a Large International Population of Patients Affected by Leber’s Hereditary Optic Neuropathy. Ophthalmology;127(5).

[17] Skorczyk-Werner A, Tońska K, Maciejczuk A, Nowomiejska K, Korwin M, Ołdak M, Wawrocka A, Krawczyński MR (2023). DNAJC30 Gene Variants Are a Frequent Cause of a Rare Disease: Leber Hereditary Optic Neuropathy in Polish Patients. Int J Mol Sci;24(24).

[18] Stenton SL, Tesarova M, Sheremet NL, Catarino CB, Carelli V, Ciara E, Curry K, Engvall M, Fleming LR, Freisinger P, Iwanicka-Pronicka K, Jurkiewicz E, Klopstock T, Koenig MK, Kolářová H, Kousal B, Krylova T, La Morgia C, Nosková L, Piekutowska-Abramczuk D, Russo SN, Stránecký V, Tóthová I, Träisk F, Prokisch H (2022). DNAJC30 defect: a frequent cause of recessive Leber hereditary optic neuropathy and Leigh syndrome. Brain;145(5).

[19] Protasoni M, Zeviani M (2021). Mitochondrial Structure and Bioenergetics in Normal and Disease Conditions. Int J Mol Sci;22(2).

[20] Danielson SR, Wong A, Carelli V, Martinuzzi A, Schapira AHV, Cortopassi GA (2002). Cells Bearing Mutations Causing Leber’s Hereditary Optic Neuropathy Are Sensitized to Fas-induced Apoptosis *. Journal of Biological Chemistry;277(8).

[21] Zorov DB, Juhaszova M, Sollott SJ (2014). Mitochondrial reactive oxygen species (ROS) and ROS-induced ROS release. Physiol Rev;94(3).

[22] Erb M, Hoffmann-Enger B, Deppe H, Soeberdt M, Haefeli RH, Rummey C, Feurer A, Gueven N (2012). Features of Idebenone and Related Short-Chain Quinones that Rescue ATP Levels under Conditions of Impaired Mitochondrial Complex I. PLOS ONE;7(4).

[23] Klopstock T, Yu-Wai-Man P, Dimitriadis K, Rouleau J, Heck S, Bailie M, Atawan A, Chattopadhyay S, Schubert M, Garip A, Kernt M, Petraki D, Rummey C, Leinonen M, Metz G, Griffiths PG, Meier T, Chinnery PF (2011). A randomized placebo-controlled trial of idebenone in Leber’s hereditary optic neuropathy. Brain;134(Pt 9).

[24] Carelli V, La Morgia C, Valentino ML, Rizzo G, Carbonelli M, De Negri AM, Sadun F, Carta A, Guerriero S, Simonelli F, Sadun AA, Aggarwal D, Liguori R, Avoni P, Baruzzi A, Zeviani M, Montagna P, Barboni P (2011). Idebenone Treatment In Leber’s Hereditary Optic Neuropathy. Brain;134(9).

[25] Catarino CB, von Livonius B, Priglinger C, Banik R, Matloob S, Tamhankar MA, Castillo L, Friedburg C, Halfpenny CA, Lincoln JA, Traber GL, Acaroglu G, Black GCM, Doncel C, Fraser CL, Jakubaszko J, Landau K, Langenegger SJ, Muñoz-Negrete FJ, Newman NJ, Poulton J, Scoppettuolo E, Subramanian P, Toosy AT, Vidal M, Vincent AL, Votruba M, Zarowski M, Zermansky A, Lob F, Rudolph G, Mikazans O, Silva M, Llòria X, Metz G, Klopstock T (2020). Real-World Clinical Experience With Idebenone in the Treatment of Leber Hereditary Optic Neuropathy. J Neuroophthalmol;40(4).

[26] Yu-Wai-Man P, Carelli V, Newman NJ, Silva MJ, Linden A, Van Stavern G, Szaflik JP, Banik R, Lubiński W, Pemp B, Liao YJ, Subramanian PS, Misiuk-Hojło M, Newman S, Castillo L, Kocięcki J, Levin MH, Muñoz-Negrete FJ, Yagan A, Cherninkova S, Katz D, Meunier A, Votruba M, Korwin M, Dziedziak J, Jurkutė N, Harvey JP, La Morgia C, Priglinger C, Llòria X, Tomasso L, Klopstock T 2024Therapeutic benefit of idebenone in patients with Leber hereditary optic neuropathy: The LEROS nonrandomized controlled trial. Cell Rep Med;5(3).

[27] Varricchio C, Beirne K, Heard C, Newland B, Rozanowska M, Brancale A, Votruba M (2020). The ying and yang of idebenone: Not too little, not too much – cell death in NQO1 deficient cells and the mouse retina. Free Radical Biology and Medicine;152.

[28] Aleo SJ, Del Dotto V, Romagnoli M, Fiorini C, Capirossi G, Peron C, Maresca A, Caporali L, Capristo M, Tropeano CV, Zanna C, Ross-Cisneros FN, Sadun AA, Pignataro MG, Giordano C, Fasano C, Cavaliere A, Porcelli AM, Tioli G, Musiani F, Catania A, Lamperti C, Marzoli SB, De Negri A, Cascavilla ML, Battista M, Barboni P, Carbonelli M, Amore G, La Morgia C, Smirnov D, Vasilescu C, Farzeen A, Blickhaeuser B, Prokisch H, Priglinger C, Livonius B, Catarino CB, Klopstock T, Tiranti V, Carelli V, Ghelli AM (2024). Genetic variants affecting NQO1 protein levels impact the efficacy of idebenone treatment in Leber hereditary optic neuropathy. Cell Reports Medicine;5(2).

[29] van Everdingen JAM, Pott JWR, Bauer NJC, Krijnen AM, Lushchyk T, Wubbels RJ (2022). Clinical outcomes of treatment with idebenone in Leber’s hereditary optic neuropathy in the Netherlands: A national cohort study. Acta Ophthalmol;100(6).

[30] Cwerman-Thibault H, Augustin S, Lechauve C, Ayache J, Ellouze S, Sahel JA, Corral-Debrinski M (2015). Nuclear expression of mitochondrial ND4 leads to the protein assembling in complex I and prevents optic atrophy and visual loss. Mol Ther Methods Clin Dev;2.

[31] Omura T (1998). Mitochondria-Targeting Sequence, a Multi-Role Sorting Sequence Recognized at All Steps of Protein Import into Mitochondria. The Journal of Biochemistry;123(6).

[32] Newman NJ, Yu-Wai-Man P, Carelli V, Moster ML, Biousse V, Vignal-Clermont C, Sergott RC, Klopstock T, Sadun AA, Barboni P, DeBusk AA, Girmens JF, Rudolph G, Karanjia R, Taiel M, Blouin L, Smits G, Katz B, Sahel J-A, Vignal C, Hage R, Catarino CB, Priglinger C, Priglinger S, Thurau S, von Livonius B, Muth D, Wolf A, Al-Tamami J, Pressler A, Schertler C, Hildebrandt M, Neuenhahn M, Heilweil G, Tsui I, Hubbard GB, Hendrick A, Dattilo M, Peragallo J, Hawy E, DuBois Med L, Gibbs D, Filho AF, Dobbs J, Carbonelli M, Di Vito L, Contin M, Mohamed S, La Morgia C, Silvestri S, Acheson J, Eleftheriadou M, Esposti S, Gemenetzi M, Leitch-Devlin L, Tucker WR, Jurkute N, SantaMaria M, Tollis H, Haller JA, Massini M (2021). Efficacy and Safety of Intravitreal Gene Therapy for Leber Hereditary Optic Neuropathy Treated within 6 Months of Disease Onset. Ophthalmology;128(5).

[33] Yu-Wai-Man P, Newman NJ, Carelli V, Moster ML, Biousse V, Sadun AA, Klopstock T, Vignal-Clermont C, Sergott RC, Rudolph G, La Morgia C, Karanjia R, Taiel M, Blouin L, Burguière P, Smits G, Chevalier C, Masonson H, Salermo Y, Katz B, Picaud S, Calkins DJ, Sahel J-A (2020). Bilateral visual improvement with unilateral gene therapy injection for Leber hereditary optic neuropathy. Science Translational Medicine;12(573).

[34] Feuer WJ, Schiffman JC, Davis JL, Porciatti V, Gonzalez P, Koilkonda RD, Yuan H, Lalwani A, Lam BL, Guy J (2016). GeneTherapy for Leber Hereditary Optic Neuropathy: Initial Results. Ophthalmology;123(3).

[35] Guy J, Feuer WJ, Davis JL, Porciatti V, Gonzalez PJ, Koilkonda RD, Yuan H, Hauswirth WW, Lam BL (2017). Gene Therapy for Leber Hereditary Optic Neuropathy: Low- and Medium-Dose Visual Results. Ophthalmology;124(11).

[36] Lam BL, Feuer WJ, Davis JL, Porciatti V, Yu H, Levy RB, Vanner E, Guy J (2022). Leber Hereditary Optic Neuropathy Gene Therapy: Adverse Events and Visual Acuity Results of All Patient Groups. Am J Ophthalmol;241.

[37] Wan X, Pei H, Zhao MJ, Yang S, Hu WK, He H, Ma SQ, Zhang G, Dong XY, Chen C, Wang DW, Li B (2016). Efficacy and Safety of rAAV2-ND4 Treatment for Leber’s Hereditary Optic Neuropathy. Sci Rep;6.

[38] Vignal-Clermont C, Girmens JF, Audo I, Said SM, Errera MH, Plaine L, O’Shaughnessy D, Taiel M, Sahel JA (2021). Safety of Intravitreal Gene Therapy for Treatment of Subjects with Leber Hereditary Optic Neuropathy due to Mutations in the Mitochondrial ND4 Gene: The REVEAL Study. BioDrugs;35(2).

[39] Biousse V, Newman NJ, Yu-Wai-Man P, Carelli V, Moster ML, Vignal-Clermont C, Klopstock T, Sadun AA, Sergott RC, Hage R, Esposti S, La Morgia C, Priglinger C, Karanja R, Blouin L, Taiel M, Sahel JA (2021). Long-Term Follow-Up After Unilateral Intravitreal Gene Therapy for Leber Hereditary Optic Neuropathy: The RESTORE Study. J Neuroophthalmol;41(3).

[40] Liu Y, Eastwood JD, Alba DE, Velmurugan S, Sun N, Porciatti V, Lee RK, Hauswirth WW, Guy J, Yu H (2022). Gene therapy restores mitochondrial function and protects retinal ganglion cells in optic neuropathy induced by a mito-targeted mutant ND1 gene. Gene Ther;29(6).

[41] Gao Y, Guo L, Wang F, Wang Y, Li P, Zhang D (2024). Development of mitochondrial gene-editing strategies and their potential applications in mitochondrial hereditary diseases: a review. Cytotherapy;26(1).

[42] Mok BY, de Moraes MH, Zeng J, Bosch DE, Kotrys AV, Raguram A, Hsu F, Radey MC, Peterson SB, Mootha VK, Mougous JD, Liu DR (2020). A bacterial cytidine deaminase toxin enables CRISPR-free mitochondrial base editing. Nature;583(7817).

[43] Cho SI, Lee S, Mok YG, Lim K, Lee J, Lee JM, Chung E, Kim JS (2022). Targeted A-to-G base editing in human mitochondrial DNA with programmable deaminases. Cell;185(10).

[44] Yi Z, Zhang X, Tang W, Yu Y, Wei X, Zhang X, Wei W (2024). Strand-selective base editing of human mitochondrial DNA using mitoBEs. Nat Biotechnol;42(3).

